# Drug monitoring in child and adolescent psychiatry for improved efficacy and safety of psychopharmacotherapy

**DOI:** 10.1186/1753-2000-3-14

**Published:** 2009-04-09

**Authors:** Claudia Mehler-Wex, Michael Kölch, Julia Kirchheiner, Gisela Antony, Jörg M Fegert, Manfred Gerlach

**Affiliations:** 1 Department of Child and Adolescent Psychiatry/Psychotherapy, University of Ulm, Steinhövelstr 5, 89075 Ulm, Germany; 2 Institute of Pharmacology of Natural Products and Clinical Pharmacology, University of Ulm, Helmholtzstr 20, D-89081 Ulm, Germany; 3 IT-Cenre, Competence Network on Parkinson's Disease, University of Marburg, Rudolf-Bultmann-Str 8, D-35039 Marburg, Germany; 4 TDM Laboratory, Department of Child and Adolescent Psychiatry, Psychosomatics and Psychotherapy, University of Wuerzburg, Fuechsleinstr 5, 97080 Wuerzburg, Germany

## Abstract

Most psychotropic drugs used in the treatment of children and adolescents are applied "off label" with a direct risk of under- or overdosing and a delayed risk of long-term side effects. The selection of doses in paediatric psychiatric patients requires a consideration of pharmacokinetic parameters and the development of central nervous system, and warrants specific studies in children and adolescents. Because these are lacking for most of the psychotropic drugs applied in the Child and Adolescent and Psychiatry, therapeutic drug monitoring (TDM) is a valid tool to optimise pharmacotherapy and to enable to adjust the dosage of drugs according to the characteristics of the individual patient. Multi-centre TDM studies enable the identification of age- and development-dependent therapeutic ranges of blood concentrations and facilitate a highly qualified standardized documentation in the child and adolescent health care system. In addition, they will provide data for future research on psychopharmacological treatment in children and adolescents, as a baseline for example for clinically relevant interactions with various co-medications. Therefore, a German-Austrian-Swiss "Competence Network on Therapeutic Drug Monitoring in Child and Adolescent Psychiatry" was founded [1] introducing a comprehensive internet data base for the collection of demographic, safety and efficacy data as well as blood concentrations of psychotropic drugs in children and adolescents.

## Introduction

Epidemiological data show remarkable differences in prescribing patterns and use of medication in child and adolescent psychiatry between the US and Europe, but also among European countries [2,3]. Prevalence of the use of antipsychotic and antidepressant medications is higher in the U.S. and prescribing patterns of substance classes differ. For example, the annual prevalence of antidepressant and stimulant medication was three times greater in the US than in the Netherlands or Germany, that of antipsychotics was 1,5–2,2 times greater, respectively [4] In the U.S. second generation antipsychotics (66% of total antipsychotic prescriptions versus 48% in the Netherlands versus 5% in Germany) and selective serotonin reuptake inhibitors (SSRIs) are the most used drugs for the respected indications, in Germany first generation antipsychotics, herbal medicines (esp. St John's wort) and tricyclic antidepressants (73% of total antidepressant prescriptions in Germany versus 48% in the Netherlands and 15% only in the U.S.) are the predominantly prescribed drugs [4]. Anyhow there is a considerable amount of off-label prescription of psychotropic medication in all western countries for the treatment of psychiatric disorders in children and adolescents. This results in a major ethical and safety problem because usual mechanisms of data collection fail. Therefore there is an increased need of generating reliable reports on individual response and especially on side effects in children and adolescents. For this reason in the U.S. large networks like the CAPTN network (Principle investigator: John March) have been created to collect so-called real life data from thousands of sites throughout the U.S. taking account of co-medication, augmentation and individual side effects. But within these networks only standardized reports are collected. There is no biological measure of serum blood-levels of the prescribed drugs nor are there any data on pharmacogenetics. In central Europe safety interest and the striving for quality assurance in medical care allows the analysis of blood samples in this context of off-label medication. Therefore it seemed to be evident that a network using standardized side effect reports comparable to the CAPTN study and collecting biological data should be built up in Germany or in Europe. In 2007/2008 we concluded a contract with Dr. March's group on the use and translation of the PAERS scale and other instruments used in the CAPTN network. At the same time we established a computer based platform for a German multi-centre observational safety-oriented study.

This paper briefly reviews the current situation in child and adolescent psychopharmacotherapy. Then it is discussed what is known about the developmental differences in physiological factors that influence the therapy with psychotropic drugs in child and adolescents. Finally, therapeutic drug monitoring (TDM), an appropriate tool for the improvement of dosing and drug safety, and the German-Austrian-Swiss "Competence Network on TDM in Child and Adolescent Psychiatry" [1] that uses a comprehensive internet data base for the collection of demographic, safety and efficacy data as well as serum levels of psychotropic drugs in children and adolescents, is described.

## Safety of psychotropic medications in children: A developmental issue

### Current situation in the psychopharmacology of child and adolescents in Europe

A widespread use of off-label medication in paediatrics, as well as in child and adolescent psychiatry characterizes the pharmacoepidemiological situation in Europe [5-7]. This situation is ethically problematic[8,9]:

• Most psychotropic drugs used in children and adolescents have neither been developed nor assessed in these age groups.

• Very few psychotropic drugs have a paediatric indication and defined posology (for example amphetamine und methylphenidate in the treatment of ADHD).

• As children and adolescents are subject to many of the same diseases as adults, and consequently often treated with the same drugs, prescribing drugs "off label" can place the paediatric population at a direct risk of under- or overdosing and a delayed risk of long-term side effects.

The deficiencies of paediatric drug development procedures and reasons for a lack of labelled drugs are well known and have been published elsewhere (e.g. [10,11]). As a result of missing data by randomized controlled trials in minors no second generation antipsychotics is labelled for minors in Germany, besides risperidone, which is only labelled for behavioural use, but not for the use in psychosis. Clozapine is labelled for minors of 16 years and older, but only as a treatment strategy in third line. The lack of evidence for the use of most SSRIs in minors implies the need for further studies on the treatment of Major Depressive Disorders in minors [12]. At present only some preparations of fluoxetine are licensed for the use of children older than eight years. Concerning SSRIs, Safer and Zito found that safety aspects of drug treatment in minors are related specifically to age groups [13,14] and pointed out the need of studies about Major Depressive Disorders in naturalistic designs to compare efficacy, effectiveness and safety of several treatment strategies under realistic conditions. Moreover, differences between the U.S. and Europe in the indicated prevalence of some disorders, e.g bipolar disorder, lead to essential disparate treatment strategies [15,16].

### Research on medications for minors in Europe

Whereas in the U.S. publicly funded clinical trials, driven by some legislation, have been conducted in the last years with high impact on clinical practise (e.g the MTA study, the TADS trial), in Europe clinical trials are predominantly driven by industry [17]. Most data about second generation antipsychotics in minors were collected by Research Units on Pediatric Psychopharmacology, driven by the "pediatric rule" [18]. Investigator initiated trials are rare in Europe [19]. A new European Directive was implemented in summer of 2007 to increase drug safety also for minors. Thus, clinical trials in minors shall be pushed with incentives, based on regulations similar to the U.S. with a sort of "stick and carrot" policy for pharmaceutical industry (e.g. extension of patents) [20]. Especially the collection of long-term safety data was one of the major aims of this directive, since most safety investigations of randomized controlled trials refer to short follow-up durations only.

### Conditions of health systems

In most European countries the health care system shows substantial differences from the U.S. Health insurance and access to care is available nearly for all people by state-run health insurance companies. Whereas this high standard is provided, on the other hand the safety aspects of drug treatment for a population with special need for protection (like minors with psychiatric disorders) are neglected. Collecting safety data by health insurances and networks for monitoring and reporting of pharmacological safety data and side effects could be easily implemented, but this did not happen yet. Especially for minors with differences in physiological factors that influence the pharmacology of drugs, a safety system in standard treatment would help to solve the black box of safety and of unknown relationship between the dosage and actions of drugs (e.g. effectiveness and side effects). At present, in Europe the ethically problematic situation of lower standards of drug-safety for children continues, due the lack of paediatric pharmacokinetic and -dynamic trials and non-existing data. The European Agency for the Evaluation of Medicinal Products (EMEA) is responsible for the implementation of the European Risk Management Strategy (ERMS). For the enhancement of safety surveillance, the introduction of a special EUDRA (European Union Drug Regulatory Authorities) Vigilance Datawarehouse and Analysis System is planned. Besides spontaneous reporting of adverse event systems, a network of centres for pharmacoepidemiology and pharmacovigilance is warranted in order to facilitate multi-centre studies or to authorize safety topics [8].

## Developmental psychopharmacology

More than 100 years ago Dr. Abraham Jacobi, the father of American paediatrics, recognised the importance of and need for age-appropriate pharmacotherapy when he wrote, "Pediatrics does not deal with miniature men and women, with reduced doses and the same class of disease in smaller bodies, but ... has its own independent range and horizon (see [21]) The recognition that developmental changes profoundly affect the responses to medications (both efficacy and side effects) produced a need for age-dependent adjustment in doses. However, the selection of doses in paediatric patients requires a consideration of pharmacokinetic parameters, and warrants specific studies in children and adolescents (See Table 1).

**Table 1:** Age-dependent factors that influence the pharmacokinetics in children and adolescents

**Pharmacokinetic parameter**	**Influencing factors**
Absorption	Gastrointestinal function (for example gastric emptying and intestinal motility; hydrochloric acid production, bile acid secretion, intestinal and body length)
Distribution	Body composition (for example extra-cellular and total-body water space, volume of distribution, changes in the composition and amount of circulating plasma proteins, body fat) Regional blood flow Organ perfusion Permeability of cell membranes Acid-base balance Passive diffusion of drugs into the central nervous system
Metabolism	Metabolic capacity (for example liver size, activity of drug-metabolising phase I and II enzymes such as P-450 cytochromes and glucuronosyltransferase)
Excretion	Renal function

(modified according to [21,42]).

### Ontogenesis of pharmacokinetics

As summarized in Table 1, developmental changes in physiology produce many of the age-associated changes in the absorption, distribution, metabolism, and excretion of psychotropic drugs following oral administration (most drugs are administered orally to children and adolescents) that result in altered pharmacokinetics and thus serve as the determinants of age-specific dose requirements. However, no systematic studies were carried out to show how each of these factors is changed over lifetime and whether there are gender dependent changes. These developmental changes in physiology have – dependent on the psychotropic drug administered – different effects on concentrations in the blood (Table 2) and most likely also at the target structures in the central nervous system (CNS). Therefore, the approach to extrapolate age-specific dosing regimes from adult data has limited value and the selection of doses in paediatric patients requires a consideration of pharmacokinetic parameters. It has been hypothesized that inaccurate dosing parameters were the reason for the negative outcome of the studies of antidepressants in paediatric patients with Major Depressive Disorder [22] (see Table 2).

**Table 2:** Effects of developmental factors on the pharmacokinetics and the efficacy of psychotropic drugs

**Developmental facto**r	**Pharmacokinetic effects**	**Clinical effects**
Liver size and activity of CYP450 enzymes ↑	Increased drug metabolism	Insufficient drug efficacy
Percentage of body fat ↓	Reduced storage of lipophilic drugs	
Glomerular filtration rate ↑	Faster urinary excretion	
Protein binding ↓	Increased drug availability	Increased risk of side effects
Gastro-intestinal resorption ↑	Increased drug availability both in peripheral organs and the brain	

CYP450, P-450 cytochrome

↑, Increase compared to adults; ↓, Decrease compared to adults

### Ontogenesis of pharmacodynamics

Although it is generally accepted that development can alter the action of and response to a drug, little information exists about the effect of human ontogenesis on interactions between psychoactive drugs and biological target structures (i.e. the pharmacodynamics) and the consequence of these interactions (i.e. efficacy and side effects). Although, cell birth, neuronal differentiation and migration of neurons to target areas are almost complete within the first few years of life in humans, there is a lifelong change in the synaptogenesis and synapse elimination with changes in the density of neurotransmitter receptors, sensitivity of signal transduction pathways, activity of neurotransmitter metabolising enzymes and density of neurotransmitter re-uptake transporters. Post-mortem studies and high-resolution structural magnetic resonance imaging longitudinal studies demonstrated non-linear region- and neurotransmitter-specific changes. For example, Giedd et al. [23] found age- and sex-specific changes in the cortical gray matter, with developmental curves for the frontal and parietal lobe peaking at about age 12 and for the temporal lobe at about age 16, whereas cortical gray matter continued to increase in the occipital lobe through age 20. The frontal and parietal gray matter peaks approximately one year earlier in females, corresponding with the earlier age of onset of puberty. In human post-mortem studies is has been shown that there is a transient elevation of both the dopamine D_1_- and D_2_- receptor density (the main therapeutic target of antipsychotics in the brain) in the early childhood; after about 2–5 years there is a rapid decline, and after ten years D_1 _and D_2 _receptor density decreases at about 3.2 and 2.2 percent per decade, respectively [24]. In addition, it has been reported that there is an age-dependent development of human neuromelanin. This dark-coloured pigment is formed in the dopamine neurons of the human midbrain and interacts with a variety of potentially damaging molecules such as iron but also with neuroleptics [25]. Neuromelanin was not present at birth and initiation of pigmentation began at approximately three years of age, followed by a period of increasing prigment granule number and increasing pigment granule colouration until age 20 [25].

The ontogenesis of the CNS has an influence on the interaction of a psychotropic drug with biological structures in the CNS (e.g. neurotransmitter metabolism, neurotransmitter receptors, neurotransmitter transporters, signal transduction) and the resulting therapeutic effect. These changes in the ontogenesis of pharmacodynamics indicate that there is a difference in the relationship between the blood concentration of a psychotropic drug and therapeutic response to a psychotropic drug in children, adolescents and adults. Indeed, we have shown by performing TDM that more than 50% of the quetiapine trough serum concentrations were not within the therapeutic range recommended for adults [26]: 40.8% of the determined values were below and 24.5% above the therapeutic range (70–170 ng/ml) recommended for adults. Interestingly, none of the patients had severe side effects.

### Pharmacogenetic aspects

Genetic variability influences drug effects from absorption of the drug until its complete elimination [27]. Genetic variability exists both at the pharmacokinetic and pharmacodynamic side of drug action. Many enzymes involved in drug metabolism carry genetic variants (polymorphisms) which can decrease enzyme activity or even lead to complete deficiency [28]. Genetic variants in drug targets such as receptor molecules or intracellular structures of signal transduction and gene regulation directly and indirectly influence drug response.

Genetic polymorphisms lead to different phenotypes of drug metabolizers which generally have been referred as "poor metabolizers" (carrying two alleles predicting a low or no enzyme activity), "intermediate metabolizers" (being heterozygous carriers of one inactive allele or of two alleles with reduced activity), and "extensive metabolizers" (carrying two active alleles) and, for some enzymes, "ultra-rapid metabolizers" (revealing a very high enzyme activity which is genetically caused by gene duplication, so far only found for CYP2D6 and CYP2A6; [29]. The phenotypes reflecting the actual enzyme activity still show high inter-individual variation especially within the intermediate and extensive metabolizer groups. Thus, genetic prediction of enzyme activity is best possible for the poor and ultra-rapid genotypes, but poor or ultra-rapid metabolizing activity can also be caused by enzyme inhibitors or inducers [30].

The prevalence of the different types of metabolizers varies a lot between ethnic groups [31]. For CYP2D6 which catalyses the hydroxylation of many tricyclic antidepressants and other psychotropic drugs, 5 to 8% poor metabolizers and 1 to 10% ultra-rapid metabolizers have been found in Caucasians. In Ethiopia and some Arab countries, even up to 30% are carriers of the CYP2D6 gene duplication [32]. CYP2C19 polymorphisms in Caucasian populations seem to be less important although several, mainly tricyclic, antidepressants are metabolized by this enzyme. In Asian populations, however, about 20% of the population are CYP2C19 poor metabolizers [33].

In young patients, especially in smaller children, pharmacogenetics might gain a special importance since the enzyme activity changes over the time especially in early development. A fatal developmental-pharmacogenetic interaction has recently been reported for codeine in a mother who genetically was an ultra-rapid metabolizer of codeine to morphine and breasted a neonate who got poisoned from morphine because of lack of glucuronidation activity which is common in neonates [34]. Thus, pharmacogenetics might gain special importance in children when enzyme activities differ from those in adults.

Pharmacogenetic testing can be a valuable tool in psychopharmacotherapy if genetic testing can be performed with a reasonable effort. Depending on the particular CYP450 enzyme, different genotyping methods are available and are offered by commercial labs, university sites or centres doing TDM. TDM and pharmacogenetic tests can advantageously be combined with TDM that to a certain extent can be considered as a phenotyping procedure. Pharmacogenetic tests might be indicated in the case of unusual plasma concentration to dose relations or when the ratio of parent substance to metabolite is distorted. Genotyping is considered as a "trait marker" and its result does not depend on environmental factors, meaning that it has only to be performed once in a person's lifetime. In general, a DNA probe is extracted from a non-centrifuged whole blood sample, but material such as buccal swabs or saliva samples may also serve. Genotyping tests are routinely available for CYP2D6 and CYP2C19, and research-based laboratories might offer further gene tests. Genetically caused variability in drug metabolism can be overcome by genotype-based dose adjustments. These dose adjustments are calculated according to the principles of bioequivalence under consideration of special circumstances like linearity of pharmacokinetics, activity of metabolites, and dose range of the underlying studies. Methods for extracting dose adjustments from pharmacokinetic data in dependence of genotypes have been developed and published elsewhere [35-37].

Pharmacogenetic testing is combined to TDM in adult drug therapy mostly for explaining abnormalities in drug metabolism, side effects, and some times therapeutic failure [38]. In children and adolescents, specific changes in enzyme activity during development lead to specific susceptibility of this patients for adverse drug effects in general, and the combination of specific developmental changes in metabolic capacity with pharmacogenetic profiles might lead to side effects or poor outcome specifically in those subgroups of patients. One study looking at newborns exposed to antidepressant treatment with SSRIs revealed that those with a low-activity genotype of the serotonin transporter had more toxicity through maternal drug therapy than those with the high-activity genotype which was reflected in lower birth weight and lower performance after birth [39]. One case of a newborn infant dying from morphine intoxication has been described due to the ultrarapid metabolizer state of the mother who metabolized codeine to morphine, and due to the low glucuronidation capacity of the infant, it got intoxicated with morphine [34]. Indeed, these are data from newborns who certainly are more susceptible to drug toxicity but we know that children and adolescents differ from adults in many aspects of drug metabolism. Thus, these patients are already at risk for over-dosing or under-dosing since we do not know their exact metabolic underpinnings. Genotyping for pharmacogenetic polymorphisms will provide additional information on the individual drug metabolizing capacity, and sometimes also information on drug efficacy, and if performed in combination with TDM, we expect further insights into the specific requirements for drug therapy of these patients.

## Aims of Therapeutic Drug Monitoring (TDM)

TDM comprises the measurement of plasma or serum levels and the documentation of both the clinical efficacy and side effects (Baumann et al., 2004). TDM is a valid tool to optimise pharmacotherapy. It enables the clinician to adjust the dosage of drugs according to the characteristics of the individual patient. The interdisciplinary TDM expert group of the Society of Neuropsychopharmacology and Pharmacopsychiatry (AGNP, [40]) analysed published data on 65 psychopharmaca and defined therapeutic ranges of plasma levels [38]. Moreover, they constituted a recommendation system for the implementation of TDM of 5 levels, indicating TDM most urgently for the treatment with lithium, but also for amitriptyline, clomipramine, clozapine, fluphenazine, haloperidole, imipramine, nortriptyline and olanzapine. For augmentation strategies or comedication in general TDM also provides support in dosage finding and prevention of toxic or unwanted side effects [38,41,42]. Other indications for TDM include the control of compliance, the lack of dosage correlated medication efficacy and the incidence of severe side effects. Because empirical data on drugs with psychotic drugs in children and adolescents are limited and most are not approved for this young age group, the administration of psychopharmaca in children and adolescents is a general indication for TDM [38,43].

## Description of the multi-centre drug monitoring data base

Because for many of the psychotropic drugs applied in the Child and Adolescent and Psychiatry data on pharmacokinetics, efficacy and side effects are lacking, and many psychiatric disorders (with exception of ADHD) have a low incidence, we established a "Competence Network on Therapeutic Drug Monitoring in Child and Adolescent Psychiatry" in December 2007 [1], including 12 Departments of Child and Adolescent Psychiatry in Germany, Austria and Switzerland. The Network uses a multi-centre TDM system including both standardized measurements of blood concentrations of psychotropic drugs and the documentation of efficacy and side effects of the medication. For practical reasons, the use of an internet data base was chosen in order to save and to systematically structure the huge data amounts that have to be expected. Such a data-based documentation will simplify final evaluation procedures. Furthermore, individuals with abnormal blood levels on the one hand or low drug efficacy or severe side effects, respectively, despite of normal plasma levels on the other hand, could be detected easily and e.g. transferred to further genetic analyses.

### Conditions

As described above, health care systems in Western Europe provide an access to care for nearly all people. Regular blood examinations that are already done as a matter of routine in clinical visits of the patients could increase data about concentrations, dose-efficacy and side-effects without bothering the patient by additional visits and enrolment in studies. This data, collected with the first aim of the individual benefit for the patient, would at the same time increase the potential benefit for this group of patients by systematically generating a database for safety parameters. The non-evidence based prescribing practise with non-systematically assessment of prescriptions, of blood levels and of effects as well as of side effects is replaced by a more evidence-based treatment with medication.

### Technical characteristics of the database

The data are recorded with the medical database system SecuTrial^®^. SecuTrial^® ^is a strictly internet-based system in connection to a relational Oracle database, made for collecting pseudonymised medical data. SecuTrial^® ^was originally developed for the Competence Network on Parkinson's disease (CNP) and adapted by the Competence Networks Dementia, Congenital Heart Defects, Creutzfeld Jakob Disease, Restless Legs Syndrome, BrainNet, European Networks-of-excellence EuroPa, EU BrainNet, the Coordination Centre for clinical trials, KKS in the last years and for clinical trials of several pharmaceutical companies. The CIO of the Competence Network on Parkinson's disease with its large experience in long-time medical registers functions as the CIO for the "Competence Network on Therapeutic Drug Monitoring in Child and Adolescent Psychiatry" as well and takes care for the register data quality according to strong scientific guidelines. SecuTrial^® ^contains functions for data input about forms, reports, statistics, inspection, export and data evaluation. The complete dataset is organised in form families. Medical data can be collected from as many research groups and as many examinations as wanted over a time line of follow-ups, presented as case history (Additional file 1, fig. 1a and fig. 1b).

To fulfil the legal requirements for data safety and protection laws of the included European countries all persons authorized for data input and view are part of a sophisticated user, privilege and role system. Patients as well as authorized persons are relocated to enclosed centres (e.g. hospitals). Due to protection of data privacy the clinical data are labelled only with a patient identification number (pseudonym) and clinical investigators can only access clinical data of their own centre. Moreover the right and role system can give authorized user access to single, some or all data forms with different view privileges. The data forms of the database system react interactive with these rights and roles: a user with a role, not authorized for a certain data form, will not be able to access.

This flexibility is used for the important interaction with the central labs. Authorized persons of the centre "central lab" may access the medication data form of all patients of all centres to enter serum levels using a second patient identification number (labID), but may not revise any other data forms.

In addition each data change is noted together with user name, date and time in the audit trail.

With its right and role system SecuTrial^® ^is certified to meet all requirements according to GCP, AMG, EMEA and FDA (21 CFR Part 11). Furthermore the system's security concept (secure hosting, firewall system, daily database and log file backups) possesses the TÜV-IT certificate "trusted site".

### Design of the platform

The platform is used for routine TDM in all cases of therapy with psychotropic drugs. For the pilot period, the study evaluation focuses on atypical antipsychotics and modern antidepressants, especially SSRIs. Every patient treated with psychotropic drugs is enrolled. Ethical committees did approve the study design stating that TDM as a part of routine procedures in order to optimize dosage finding and prevention of side effects even needs no informed consent. However, for data safety reasons, forms of informed consent on data collection and data analysis is provided nonetheless.

Due to standardized operational procedures, 10–12 hours after the last dose, blood samples (7.5 mL) are collected in the morning at steady state. Analyses are performed in centralized labs providing standardized procedures by high-performance liquid chromatography (HPLC) and ultraviolet (UV) detection according to the guidelines of the AGNP TDM expert group.

The database, using codes for pseudonymization, comprises demographic data (age, gender, body weight, height, BMI, diagnose, medication given, dosage, beginning of therapy with this medication, pattern of titration before, use of nicotine or alcohol or other interfering substances, compliance) and validated tools on the general efficacy of medication and the patient's global functioning as well as the documentation of side effects. Specific rating scales for schizophrenia and depressive disorders are implemented already, further instruments might be included depending from the scientific aims. The Pediatric Adverse Events Rating Scale (PAERS), developed for the Child and Adolescent Psychiatry Trials Network (CAPTN; [44]), is provided for adverse events. Rater trainings are implemented within initiation meetings of participating centres and are repeated regularly when additional clinical investigators are enrolled to warrant a highly qualified standardized documentation. In addition, the principal investigators of each centre are obliged to train their assistants.

For every patient enrolled, a baseline visit is performed comprising the demographic items and psychometric tools as described above. As soon as the new medication is started, follow-up visits including serum level analyses report on the efficacy and safety of the psychopharmacological treatment regarding potential interfering aspects. TDM is performed until adequate remission of the target symptom of medication.

## Perspectives

Adequate dosing in paediatric psychiatric patients requires a consideration of pharmacokinetic parameters and the development of the CNS, and warrants specific studies in children and adolescents. Because these data are lacking for most of the psychotropic drugs applied in the Child and Adolescent and Psychiatry, TDM is a valid tool to optimise drug therapy and to enable to adjust the dosage of drugs according to the characteristics of the individual patient. Multi-centre TDM studies providing large patient samples, standardized measurements of blood concentrations of psychotropic drugs, baseline and follow-up assessment of psychopathology and the documentation of side effects enable the identification of age- and development-dependent therapeutic ranges of blood concentrations, thus facilitating dosage finding, improving efficacy and minimizing the risk of side effects. Pharmacokinetic abnormalities in individual patients could be further investigated by the classification of pharmacogenetic subtypes. Moreover, multi-centre standardized TDM documentation will provide data for future research on psychopharmacological treatment in children and adolescents, as a baseline, for example, for clinically relevant interactions with various co-medications. A TDM data base therefore will not only increase the limited knowledge on pharmacokinetic and pharmacodynamic conditions in minors but also provide individual benefits for the patients participating in terms of individualized therapy with psychotropic drugs. Moreover, it facilitates a highly qualified, standardized documentation in the child and adolescent health care system.

From a scientific perspective, the TDM database can be regarded as a basic tool for the implementation of multi-centre clinical trials and observational studies since the body of data collection can be extended or changed flexibly, depending on the aims of the respective study. SecuTrial offers monitoring and query systems, data entry complete functions, source data verification functions and AE/SAE forms thus providing any technical equipment that is required for trial support. Additional investigations might either be set-up independently from the original data base or be implemented within the existing TDM register. Therefore, on the one hand, it allows the restriction on basic data only for centres that primarily want to focus on essential clinical data documentation, and it enables scientific trials with specifically increased data collections on the other hand.

## Competing interests

In the last 4 years since the department in Ulm was founded JMF received unrestricted research grants from State and national governmental organizations and from the Volkswagen foundation, the Eberhardt foundation, from Eli Lilly Foundation, from Janssen and from Celltech/USB. JMF was involved in clinical trials with Janssen, Medice, Lilly, AstraZeneca, and serves on a DSMB for Pfizer.

JMF got travel grants from or served as a consultant for Aventis, Bayer, Bristol-MS, J&J, Celltech/USB, Lilly, Medice, Novartis, Pfizer, Ratiopharm, Sanofi-Synthelabo; VFA & Generikaverband, the Vatican, NIMH, AACAP, DFG, EU and European Academy. JMF states they he has no shares and no direct affiliation with a pharmaceutical company. The other authors declare that they have no competing interests.

## Authors' contributions

CMW is principal investigator of the TDM database (conception and design) and chair of the "Competence Network of Therapeutic Drug Monitoring in Child and Adolescent Psychiatry". JMF is the head of the commission developmental psychopharmacology of the three German professional societies and initiated the network based on decisions of the commission. MG (head of the TDM laboratory in Würzburg) was involved in the design of the database, especially concerning laboratory items. GA performed the data base setup. JK is responsible for the design of the pharmacogenetic part. MK, JMF, CMW and MG contributed substantially to the drafting of the manuscript. GA drafted the methods part. JK drafted the pharmacogenetic part of the paper. All of the authors read and approved the final manuscript.

## Supplementary Material

Additional file 1 **Additional figures**. **Figure 1a: **Figure 1 Form overview patient register TDM database. **Figure 1b: **Link SecuTrial^® ^(TDM database). **Figure 2: **screenshot1 of TDM database. **Figure 3: **screenshot2 of TDM database. **Figure 4: **screenshot3 of TDM database. Click here for file
