# Supplementary material for: Drug monitoring in child and adolescent psychiatry for improved efficacy and safety of psychopharmacotherapy
Source: Child Adolesc Psychiatry Ment Health. 2009 Apr 9;3:14. doi: 10.1186/1753-2000-3-14 (PMC2674035; doi:10.1186/1753-2000-3-14)

## Additional file 1

## Figure 1a


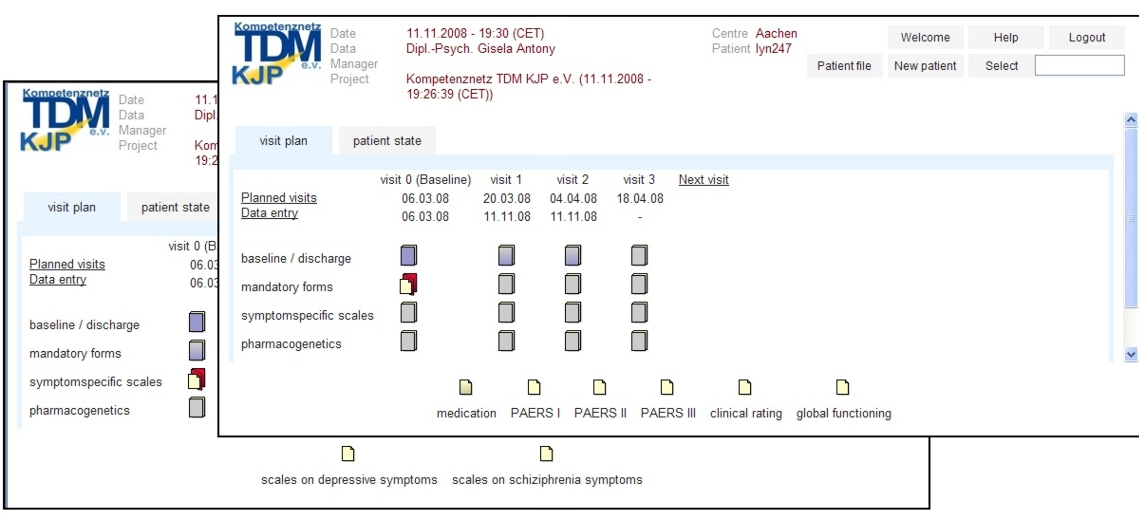


## Figure 1b

SecuTrial® consists of five modules, each with own URL address and own access authorisation: Task Daemon (generation of statistics, message transmission), Form Builder (construction and configuration of projects and registers, form generating, internal database adaptation), Admin Tool (user management, management of rights and roles), Data Capture (creation of medical data sets for new patients, data entry, data change) and Export Search Tool (search for data, data export). The application is programmed in Java 2 SE and implemented for the WebObjects-Application Server. Two different frameworks are used: one for server side generation of web pages and one, which represents the object-relational model of the tables in the database and controls the data access. The application logic is related to a web-session. The GUI is representing HTML-pages with dynamic data bindings. The underlying database is implemented in SQL with ORACLE-specific extensions. The software development was strictly proceeded in accordance to a standardized procedural model, meeting all ISPE GAMP4 requirements of software validation.

# Figure 2


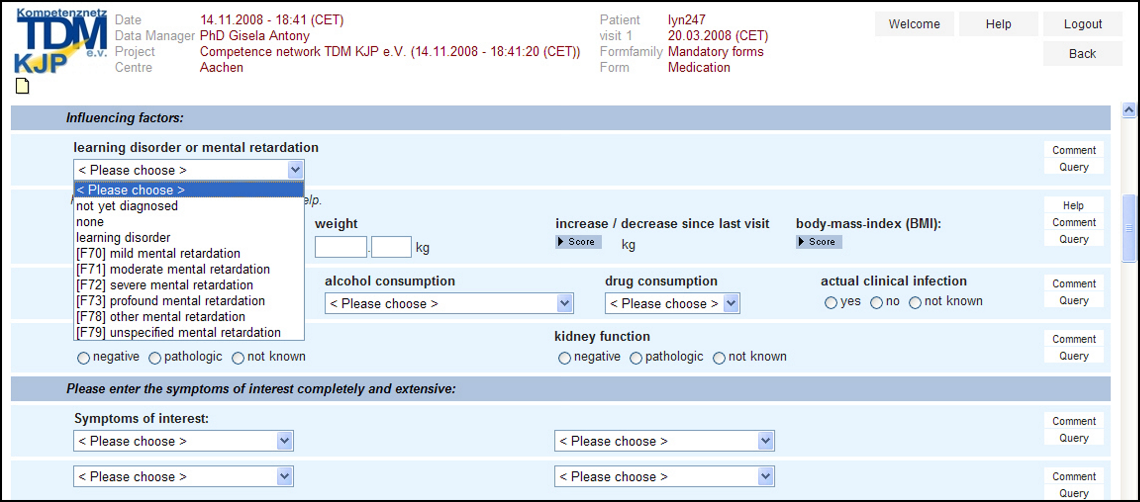


# Figure 3


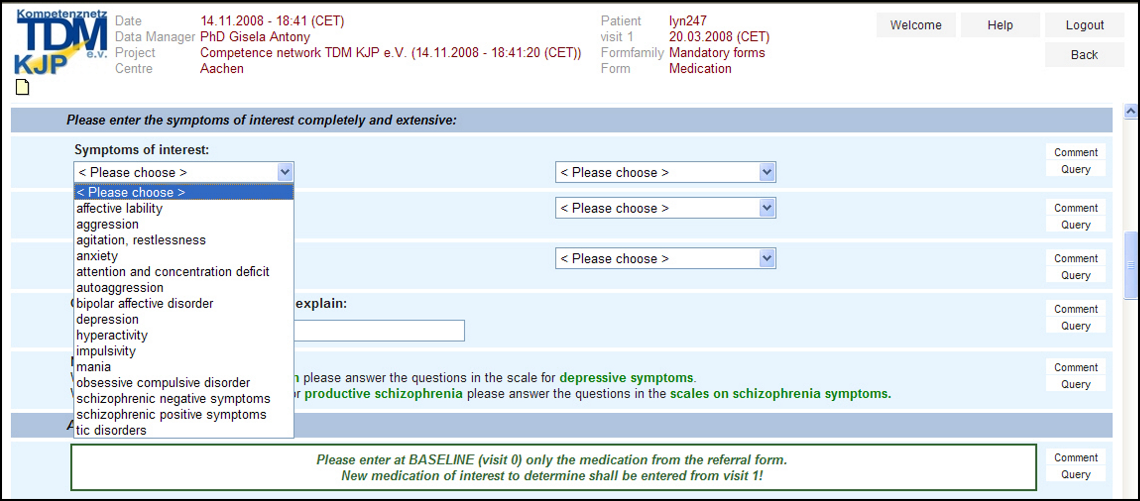


# Figure 4


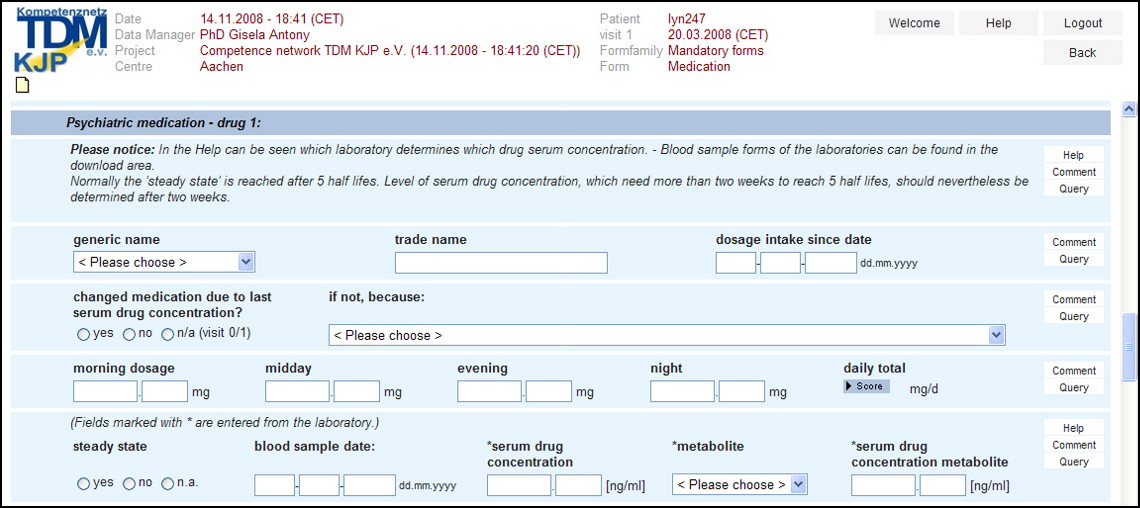

Supplement: Additional file 1 — Additional figures. Figure 1a: Figure 1 Form overview patient register TDM database. Figure 1b: Link SecuTrial® (TDM database). Figure 2: screenshot1 of TDM database. Figure 3: screenshot2 of TDM database. Figure 4: screenshot3 of TDM database. [file 1753-2000-3-14-S1.doc]
